#  Values of mean platelet volume in patients with chronic tonsillitis and adenoid hypertrophy

**DOI:** 10.12669/pjms.292.2715

**Published:** 2013-04

**Authors:** Cevik Cengiz, Yengil Erhan, Tutanc Murat, Akbay Ercan, Silfeler Ibrahim, Gulmez Ihsan, Akoglu Ertap

**Affiliations:** 1 Cevik Cengiz, MD, Assistant Professor, Departments of Otolaryngology, Mustafa Kemal University School of Medicine, Antakya, Turkey.; 2 Yengil Erhan, MD, Assistant Professor, Departments of Family Medicine, Mustafa Kemal University School of Medicine, Antakya, Turkey.; 3 Tutanc Murat, MD, Assistant Professor, Departments of Pediatrics, Mustafa Kemal University School of Medicine, Antakya, Turkey.; 4 Akbay Ercan, MD, Assistant Professor, Departments of Otolaryngology, Mustafa Kemal University School of Medicine, Antakya, Turkey.; 5 Silfeler Ibrahim, MD, Assistant Professor, Departments of Pediatrics, Mustafa Kemal University School of Medicine, Antakya, Turkey.; 6 Gulmez Ihsan, MD, Assistant Professor, Departments of Otolaryngology, Mustafa Kemal University School of Medicine, Antakya, Turkey.; 7 Akoglu Ertap, MD, Associate Professor, Departments of Otolaryngology, Mustafa Kemal University School of Medicine, Antakya, Turkey.

**Keywords:** Chronic tonsillitis, Obstructive sleep apnea, Mean platelet volume

## Abstract

***Objectives:*** Chronic tonsillitis (CT)-adenoid hypertrophy (AH) is the most common cause of obstructive sleep apnea (OSA), which is one of the most common reasons of nocturnal hypoxia in children. However, there is limited information about the relationship between childhood OSA and atherosclerosis or cardiac diseases. In the present study, we evaluated the relationship between mean platelet volume (MPV) and CT-AH which is the most frequent cause leading OSA in children.

***Methodology***: The medical records of 200 children, who underwent adenoidectomy or adenotonsillectomy with a diagnosis of adenoid hypertrophy and/or chronic tonsillitis between October, 2010 and June, 2012, and 240 healthy controls were evaluated. Subjects were classified into 3 groups. Group I consisted of patients who underwent adenoidectomy, whereas Group II consisted of patients who had adenotonsillectomy. Healthy children were employed as control group. White blood cell count (WBC), platelet count (PLT), hemoglobin (Hb) levels and mean platelet volume (MPV) values were recorded individually.

***Results:*** MPV values were 6.6±0.8, 6.6±0.7 and 7.3±0.9 in Group I, Group II and control group, respectively. It was found that MPV values in groups I and II were significantly lower than control group. There was no significant difference between group I and II.

***Conclusion*** **:** Obstructive sleep apnea (OSA) caused by CT-AH is associated with low MPV values in childhood.

## INTRODUCTION

Adenoid-tonsil localized at oropharynx and nasopharynx is a component of Waldeyer’s tonsilar ring. IgA secreted by this lymphoid tissue has an important role in mucosal defense. In children, tonsillitis causes fever, sore throat, dysphagia, while associated adenoid hypertrophy causes snoring, sleep with open mouth and nocturnal apnea. In children, persisting obstruction findings may cause aggressive behavior, anxiety, impaired attention, depression, somatization disorders and growth retardation at long-term.^1^^,^^2^

The treatment is adenotonsillectomy in this psychosocial and medical problem. In USA, it is the most common operation performed at childhood period.^1^ Chronic tonsillitis can cause severe stress and growth retardation as well as symptoms including sleep disorders, snoring, sleep with open mouth, dysphagia and poor appetite.^2^ The etiopathogenesis of growth retardation isn’t fully understood. However, implied factors include poor appetite and dysphagia causing low caloric intake, nocturnal hypoxemia and acidosis, and higher energy expenditure caused by increased respiratory effort.^3^

Mean platelet volume (MPV) is a parameter used as a platelet activation marker. MPV that is related to function and activation of platelets has been used as a marker of atherosclerosis.^4^ Various studies have suggested that there is an increased risk for atherosclerosis and cardiac diseases in patients with adult form of obstructive sleep apnea.^5^^-^^7^ There is limited numbers of studies which found that MPV values, considered as a marker of atherosclerosis, were elevated in adult patients with OSA.^8^

In children, CT-AH is most frequent cause of OSA that is one of the reasons leading nocturnal hypoxia. However, data is limited regarding relationship between childhood OSA and atherosclerosis or cardiac diseases.^6^ In our study, we evaluated relationship between MPV and CT-AH which is most frequent cause that leads OSA in children.

## METHODOLOGY

Two-hundred children who underwent adenoidectomy or adenotonsillectomy with a diagnosis of AH and/or CT between October, 2010 and June, 2012 in Department of Otolaryngology at Mustafa Kemal University, Medicine School, were included in this study. Age-matched 240 controls without symptoms of upper respiratory tract obstruction, systemic disease, acute/chronic infection or disease presented to pediatrics and family medicine outpatient clinics were also reviewed. Patients included in this study were assessed in three groups. Group I (n=92) consisted of patients who underwent adenoidectomy, whereas Group II (n=108) consisted of those who had adenotonsillectomy. Healthy children comprised control group (n=246). In our clinic, tonsillectomy is performed to children (≥3 years of age) with an attack frequency of seven or more in a year, five or more per year for two years, or three or more per year for three years. Adenoidectomy was performed in patients with adenoid vegetation causing 85-90% or more obstruction in nasopharynx. 

Venous blood samples were taken into tubes containing EDTA. ABBOTT CELL DYN 3700 (ABBOTT PARK IL 60064 USA), auto analyzer was used for total blood count analyzed in the central laboratory of our hospital. White blood cell (WBC), platelet (PLT), hemoglobin (Hb) and MPV were individually assessed.

Gender and age distribution were considered in patients who met inclusion criteria, of the 92 patients in group one, 53 (57.6%) were boys and 39 (42.4%) were girl, whereas 65 (60.2%) were boys and 43 (39.8%) were girls of the 108 patients in group two. Of the 246 controls, 126 (51.2%) were boys and 120 (48.8%) were girls. Mean age was 8.08±3.76 (min:2, max:18), 6.86±2.82 (min:3, max:15) and 8.01±4.33 (min:2, max:18) years in group 1, group 2 and control group, respectively.

***Statistical Analysis: ***SPSS for Windows version 13.0 (Statistical Package for Social Sciences) were used for statistical analysis. Numeric data obtained by measurement were expressed as arithmetic average and standard deviation, while categorical data obtained with counting as number and percentage. One-way ANOVA and non-parametric Kruskall-Wallis test were used. Data found to be significant in Pearson correlation test among continuous variables and correlation test were analyzed in multivariate linear regression model. P<0.05 was considered as significant for all statistical data.

## RESULTS

There was no significant difference between groups in terms of age and gender (p=0.89 and p=0.24). Hb levels were 12.23±0.94 g/dL in group 1; 12.08±0.92 g/dL in group 2; and 12.15±1.15 g/dL in control group. There was no significant difference in Hb values among groups (p=0.256). WBC counts were 8.30±1.81×10^3^/μL in group 1; 8.41±1.93x10^3^/μL in group 2; and, 8.76±2.82×10^3^/μL in control group. There was no significant difference in WBC counts among groups (p═0.914).

PLT counts were 353±65×10^3^/μL in group 1; 345±70 ×10^3^/μL in group 2; and 307±87×10^3^/μL in control group. A significant difference was found in PLT counts between control group compared to group 1 and 2 (p<0.001), whereas there was no significant difference between groups 1 and 2 (p═0.719). There was a significant relationship between PLT counts and Hb, WBC, age or MPV values, where PLT counts were increased by increasing age, Hb levels and WBC counts. On contrary, it was decreased by increasing MPV values (p<0.001).

MPV values were 6.6±0.8 fL (min: 4.8, max: 9.0) in group 1; 6.6±0.7 fL (min: 5.3, max: 8.7) in group 2; and 7.3±0.9 fL (min: 5.2, max: 10.8) in control group. A significant difference was found in MPV values between control group and group 1 or 2 (p<0.001). There was a significant relationship between MPV values and age or PLT counts (p<0.001). MPV values were increased by advancing age, while decreased by increasing PLT counts (Table 1). There was no significant relationship between MPV values and Hb or WBC counts (p═0.403 and p═0.361, respectively).

**Fig.1:**
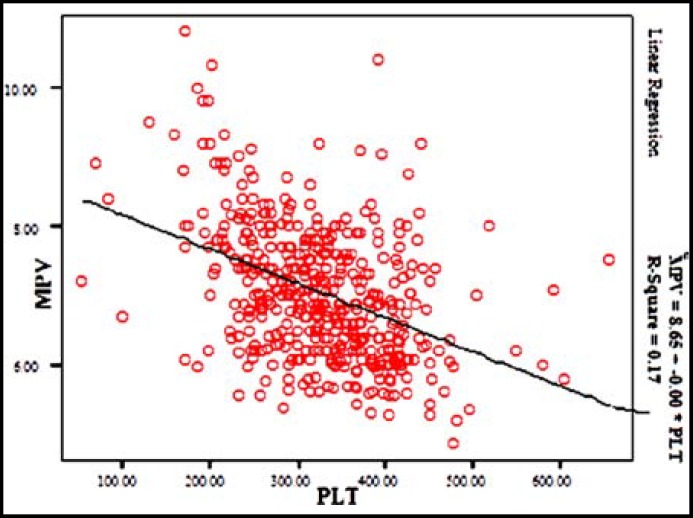
Linear Regregision Analysis MPV and PLT

When correlation test was performed on continuous variables, it was found that MPV values were positively correlated with age (r=0.270; p<0.001), while negatively correlated to PLT counts (r=-0.417; p<0.001) (Fig.1; Table-II).

**Table-I:** Relationship among MPV-PLT between the groups

*Parameter*	*Group 1* *(n:92)*	*Group 2* *(n:108)*	*Control* *(n:246)*	*Group comparisons* *(p value)* ^*^
MPV (fL)	6.6±0.8	6.6±0.7	7.3±0.9	1–2 (1.00), 1–3 (0.001), 2–3 (0.001)
PLT (×103/μL)	353±65	345±70	307±87	1–2 (0.71), 1–3 (0.001), 2–3 (0.001)
				

**Table-II:** Linear Regregision Analysis of Factors Effecting MPV

	*Unstandardized Coefficients*		*Standardized Coefficients*	*t*	*p*
	*B*	*Std. Error*	*Beta*		
Constant	9.100	.535		17.010	.000
PLT	-.005	.001	-.421	-9.699	.000
AGE	.071	.012	.292	6.124	.000
WBC	.054	.017	.140	3.139	.002
HGB	-.120	.041	-.133	-2.907	.004

## DISCUSSION

CT-AV is a disease that can cause snoring, sleep with open mouth, OSA episodes; and in addition, it can cause orofacial anomaly, poor appetite, growth retardation, aggressive behavior, anxiety, impaired attention, depression and somatization disorders at long-term, if it is not treated.^1^^,^^4^ In children, CT-AV which causes nocturnal hypoxia is the most frequent cause of OSA. ^9^^,^^10^ In the present study, contrary to adults, we found that MPV values were significantly decreased in children with CT-AV when compared to controls.

In the adult literature, there are several articles regarding association of MPV to atherosclerosis.^5^^-^^7^ However, best to our knowledge, limited numbers of studies exist in literature suggesting a relationship between MPV and atherosclerosis in children.^11^^,^^12^ There are studies showing a relationship between atherosclerosis and obesity in children, although there is no such study in our patient population.^13^ Atherosclerosis formation in children is affected by various factors including genetic and environmental factors, diet and obesity.^14^

In a study by Varol et al., it was reported that MPV values were significantly increased in adults with OSA compared to control group.^8^ In a further study in the same cohort, they found that MPV values were decreased after 6-months CPAP therapy.^15^ Risks of atherosclerosis and cardiac disease are increased in adult patients with OSA.^5^^-^^7^ It was reported that MPV value was increased in patients with OSA, which is in line with increased risk for atherosclerosis and cardiac disease.^8^ On the other hand, there are studies in which increased MPV values were detected in acute myocardial infarction.^11^^,^^16^^,^^17^ In a study by Leader et al., it has been suggested that MPV is an important marker in thrombosis.^18^

In a study on children with asthma by Tuncel et al., no significant difference was found in MPV values among controls, children with asthmatic crisis and those in remission.^19^ In a study on patients with chronic obstructive pulmonary disease (COPD), Ulasli et al reported that MPV values were significantly decreased during episodes, but not significantly differ from control group during stable period.^20^ In our study, we found decreased MPV values in children with CT-AV that leaded to obstruction of upper respiratory tract.

Furthermore, MPV values were found to be significantly lower than control group in a study on children with acute appendicitis.^21^ We failed to detect a significant difference for concurrent AV and CT. MPV value was affected by PLT count and age. MPV value was increased by increasing age, while decreased by increasing PLT count. In the linear regression analysis, we found that decreased MPV values in patients with CT-AV and AV was independent from age and PLT count.

Increased MPV value in adult form of OSA has been attributed to increased risk of atherosclerosis.^8^^,^^15^ Contrary to adults, we detected that MPV values were low in children with symptoms of upper respiratory tract obstruction. According to our results, CT-AV is resulted in OSA but not lead increased MPV values. There is a negative correlation between OSA and MPV value in children.

## CONCLUSION

To reveal the risk of atherosclerosis at childhood, one should not depend on MPV alone. There is a need for further studies which will evaluate risk factors such as obesity or diet collectively.

## References

[1] Mitchell RB, Kelly J (2005). Child behavior after adenotonsillectomy for obstructive sleep apnea syndrome. Laryngoscope.

[2] Flanary VA (2003). Long-term effect of adenotonsillectomy on quality of life in pediatric patients. Laryngoscope.

[3] Martin JF, Trowbridge EA, Salmon G, Plumb J (1983). The biological significance of platelet volume: its relationship to bleeding time, platelet thromboxane B2 production and megakaryocyte nuclear DNA concentration. Thromb Res.

[4] Gigante J (2005). Tonsillectomy and adenoidectomy. Pediatr Rev.

[5] Drager LF, Polotsky VY, Lorenzi-Filho G (2011). Obstructive sleep apnea: an emerging risk factor for atherosclerosis. Chest.

[6] Hill CM, Hogan AM, Onugha N, Harrison D, Cooper S, McGrigor VJ (2006). Increased cerebral blood flow velocity in children with mild sleep-disordered breathing: a possible association with abnormal neuropsychological function. Pediatrics.

[7] Li RC, Haribabu B, Mathis SP, Kim J, Gozal D (2011). Leukotriene B4 receptor-1 mediates intermittent hypoxia-induced atherogenesis. Am J Respir Crit Care Med.

[8] Varol E, Ozturk O, Gonca T, Has M, Ozaydin M, Erdogan D (2010). Mean platelet volume is increased in patients with severe obstructive sleep apnea. Scand J Clin Lab Invest.

[9] Izu SC, Itamoto CH, Pradella-Hallinan M, Pizarro GU, Tufik S, Pignatari S (2010). Obstructive sleep apnea syndrome (OSAS) in mouth breathing children. Braz J Otorhinolaryngol.

[10] Muzumdar HV, Sin S, Nikova M, Gates G, Kim D, Arens R (2011). Changes in heart rate variability after adenotonsillectomy in children with obstructive sleep apnea. Chest.

[11] Bilginer Y, Ozaltin F, Basaran C, Duzova A, Besbas N, Topaloglu R (2008). Evaluation of intima media thickness of the common and internal carotid arteries with inflammatory markers in familial Mediterranean fever as possible predictors for atherosclerosis. Rheumatol Int.

[12] Makay B, Turkyilmaz Z, Unsal E (2009). Mean platelet volume in children with familial Mediterranean fever. Clin Rheumatol.

[13] Barton M (2012). Childhood obesity: a life-long health risk. Acta Pharmacol Sin.

[14] Turck D (2011). Childhood diet and cardiovascular risk factors. Bull Acad Natl Med.

[15] Varol E, Ozturk O, Yucel H, Gonca T, Has M, Dogan A, Akkaya A (2011). The effects of continuous positive air way pressure therapy on mean platelet volume in patients with obstructive sleep apnea. Platelets.

[16] Kilciler G, Genc H, Tapan S, Ors F, Kara M, Karadurmus N (2010). Mean platelet volume and its relationship with carotid atherosclerosis in subjects with non-alcoholic fatty liver disease. Ups J Med Sci.

[17] Ozkan B, Uysal OK, Duran M, Sahin DY, Elbasan Z, Tekin K Relationship Between Mean Platelet Volume and Atherosclerosis in Young Patients With ST Elevation Myocardial Infarction. Angiology.

[18] Leader A, Pereg D, Lishner M (2012). Are platelet volume indices of clinicaluse? A multidisciplinary review. Ann Med.

[19] Tuncel T, Uysal P, Hocaoglu AB, Erge DO, Karaman O, Uzuner N (2012). Change of mean platelet volume values in asthmatic children as an inflammatory marker. Allergol Immunopathol (Madr).

[20] Ulasli SS, Ozyurek BA, Yilmaz EB, Ulubay G (2012). Mean platelet volume: an inflammatory marker in acute exacerbation of chronic obstructive pulmonary disease. Pol Arch Med Wewn.

[21] Bilici S, Sekmenli T, Goksu M, Melek M, Avci V (2011). Mean platelet volume in diagnosis of acute appendicitis in children. Afr Health Sci.

